# Rhizosphere Microbial Effects on Soil Quality of *Pinus massoniana* and *Schima superba* Mixed Plantations

**DOI:** 10.3390/plants15101482

**Published:** 2026-05-12

**Authors:** Wenyue Wang, Wei Yang, Wenqing Song, Shengyi Huang, Jianming Lai, Zhichun Zhou, Pengcheng Wang, Bin Wang

**Affiliations:** 1 College of Horticulture and Forestry Sciences, Huazhong Agricultural University, Wuhan 430000, China; wwy19970424@163.com (W.W.);; 2 Research Institute of Subtropical Forestry, Chinese Academy of Forestry, Hangzhou 311400, China; 3 Mingxi State-Owned Forest Farm, Sanming 365200, China

**Keywords:** *Pinus massoniana*, *Schima superba*, mixed forest, rhizosphere microorganism, nutrient cycling

## Abstract

This study aimed to reveal the rhizosphere microbial community structure, carbon–nitrogen–phosphorus (C-N-P) nutrient cycling processes, and functional gene characteristics of *Pinus massoniana* and *Schima superba* in mixed forests. Furthermore, we sought to elucidate the microbial mechanisms by which mixed-species afforestation enhances soil quality improvement, providing a theoretical basis in soil microbiology for the cultivation of these mixed forests. The research subjects included pure *P. massoniana* plantations (CLPs), pure *S. superba* plantations (CLSs), and individual *P. massoniana* (HJP) and *S. superba* (HJS) trees within mixed plantations (HJLs). We collected rhizosphere and bulk soil samples to analyze their physicochemical properties and enzyme activities. Metagenomic sequencing was employed to profile the rhizosphere microbial communities and functional genes involved in C-N-P cycling. Furthermore, by integrating a functional gene co-occurrence network analysis with structural equation modeling (SEM), we systematically elucidated the coupling relationships among the stand types, soil properties, microbial communities, and nutrient cycling. Mixed planting significantly improved soil quality; compared to the CLP and CLS forests, the nitrate nitrogen (NO_3_^−^-N) content in the mixed forest soils increased by 121.01% and 120.10% (*p* < 0.05), and the activity of urease (URE) also significantly increased by 123.99% and 49.56%, respectively. Mixing significantly altered the microbial community structure. In the bacterial community of the mixed forests, the abundance of nitrogen-fixing and potentially phosphorus-solubilizing bacteria from the genera *Paraburkholderia* and *Burkholderia* increased. In the fungal community, the arbuscular mycorrhizal fungus *Rhizophagus*, which possesses a nutrient absorption advantage, exhibited absolute dominance, with its relative abundance ranging from 14.84% to 88.81%. The abundances of genes associated with denitrification and phosphorus starvation regulation were significantly upregulated in the mixed forests; notably, the abundance of phosphorus starvation regulation genes in the HJSs was 18.84% higher than that in the CLSs. A co-occurrence network analysis demonstrated that the proportion of positive correlation edges in the HJP nitrogen cycling network reached as high as 75.0%, and the average degree of the HJS phosphorus cycling network (2.691) surpassed that of the CLSs. The structural equation modeling further revealed that the association strength between the fungi and phosphorus cycling genes in the mixed forests increased to R^2^ = 0.915 (*p* < 0.01) from R^2^ = 0.213 in the pure forests. This mixed planting practice transforms nutrient cycling from a resource-competitive mode to a microbially synergized mode, thereby forming an efficient endogenous nutrient cycling system. This synergistic rhizosphere microbial effect is a key internal mechanism for overcoming nutrient bottlenecks and should serve as a diagnostic indicator of soil recovery in the ecological restoration of degraded pine forests.

## 1. Introduction

The establishment of mixed-species plantations is a crucial strategy to overcome the ecological degradation often associated with monoculture forests. By optimizing resource allocation through interspecific niche complementarity, mixed forests enhance ecosystem services and significantly ameliorate the soil microenvironment [1,2,3]. The introduction of heterogeneous litter and diverse root exudates mitigates soil acidification and expands the carbon and nitrogen pools, thereby accelerating soil nutrient cycling. Throughout these belowground biogeochemical processes, soil microorganisms act as the primary engines, playing an irreplaceable role in driving nutrient turnover and sustaining soil fertility in mixed plantations [4,5,6].

Previous studies have demonstrated that coniferous–broadleaved mixtures can stimulate microbial activity via the rhizosphere priming effect, increasing the microbial biomass and reshaping the taxonomic composition [7,8]. Increased tree species diversity expands the niche space, promoting mutualistic taxa, such as arbuscular mycorrhizal fungi (AMF) [9,10,11]. However, most of the existing research has primarily focused on these taxonomic shifts or isolated changes in functional gene abundance [12]. A systematic understanding of how microbial communities respond to tree species mixing through the synergistic expression of C-N-P cycling genes remains limited [13]. While gene abundance indicates metabolic potential, the topological structures of functional gene co-occurrence networks (e.g., modularity and positive/negative associations) reveal critical emergent properties, such as metabolic coordination and synergistic utilization strategies, which cannot be inferred from the abundance data alone. Understanding these network interactions is essential for revealing how ecosystem stability is maintained under heterogeneous resources [14].

*Pinus massoniana* is a major afforestation species in subtropical China; however, long-term pure plantation management has led to severe soil acidification, nutrient depletion, and a decline in microbial functional diversity [15]. To mitigate these issues, *Schima superba*—characterized by rapid growth, nutrient-rich litter, and natural fire resistance—is widely planted alongside *P. massoniana* [16]. Although the macroscopic benefits of this mixed-planting pattern are well documented, it remains largely unclear how the introduction of *S. superba* reshapes the rhizosphere microbiome [17]. Specifically, the mechanisms by which this mixture constructs efficient C-N-P functional gene interaction networks to alleviate native nutrient turnover bottlenecks remain unresolved [18,19].

To address these knowledge gaps, we utilized metagenomic sequencing and network analysis to untangle the multidimensional coupling mechanisms among stand types, soil properties, and rhizosphere microbiomes in pure *P. massoniana,* pure *S. superba*, and mixed plantations. Our specific objectives were to determine how mixed planting alters the microbial community structure and to verify whether it constructs more highly connected functional networks. We hypothesized that: (1) tree species mixing significantly increases the abundance of key nutrient cycling genes and strengthens their synergistic associations; and (2) by forming highly modular and connected functional gene networks, mixed plantations shift microbial metabolism from competition to synergy, effectively overcoming the nutrient turnover bottlenecks inherent in pure stands.

## 2. Results

### 2.1. Differences in Bulk Soil Properties Among Pure and Mixed Plantations

**Figure 1:**
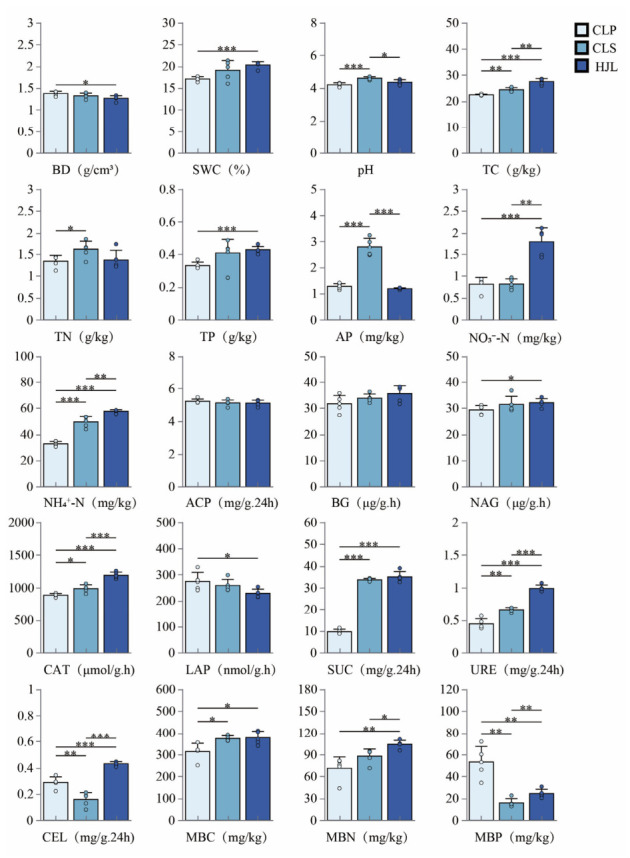
Differences in non-rhizosphere soil properties among different forest types. Note: BD: bulk density; SWC: soil water content; TC: total carbon; TN: total nitrogen; TP: total phosphorus; AP: available phosphorus; NO_3_^−^-N: nitrate nitrogen; NH_4_^+^-N: ammonium nitrogen; ACP: acid phosphatase; BG: β-glucosidase; NAG: N-acetyl-β-D glucosaminidase; CAT: catalase; LAP: leucine aminopeptidase; SUC: sucrase; URE: urease; CEL: cellulase; MBC: microbial biomass carbon; MBN: microbial biomass nitrogen; MBP: microbial biomass phosphorus. *, *p* < 0.05; **, *p* < 0.01; ***, *p* < 0.001.

Mixed planting of *P. massoniana* and *S. superba* significantly improved the soil quality (Figure 1). The contents of soil organic carbon, nitrate nitrogen, and ammonium nitrogen in the mixed plantations (HJLs) were 22.73%, 121.01%, and 75.48% higher than those in the pure *P. massoniana* plantation (CLP), and 12.88%, 120.10%, and 16.27% higher than those in the pure *S. superba* plantation (CLS), respectively (*p* < 0.01). Furthermore, the activities of soil enzymes related to carbon cycling were significantly enhanced in the mixed stands; specifically, sucrase (SUC) and cellulase (CEL) activities were 264.52% and 49.71% higher than in the CLP, and 3.95% and 170.44% higher than in the CLS (*p* < 0.01). Regarding nitrogen cycling, urease (URE) activity in the mixed plantations increased significantly, being 123.99% and 49.56% higher than in the CLP and CLS, respectively (*p* < 0.01). The contents of microbial biomass carbon (MBC) and nitrogen (MBN) in the mixed stands were significantly higher than those in the CLP, while the microbial biomass phosphorus (MBP) was significantly lower than in the CLP (*p* < 0.05). This indicates that mixed planting mitigates the microbial immobilization of phosphorus observed in pure pine stands, thereby facilitating the regulation of phosphorus bioavailability.

### 2.2. Differences in Rhizosphere Microbial Community Composition

**Figure 2:**
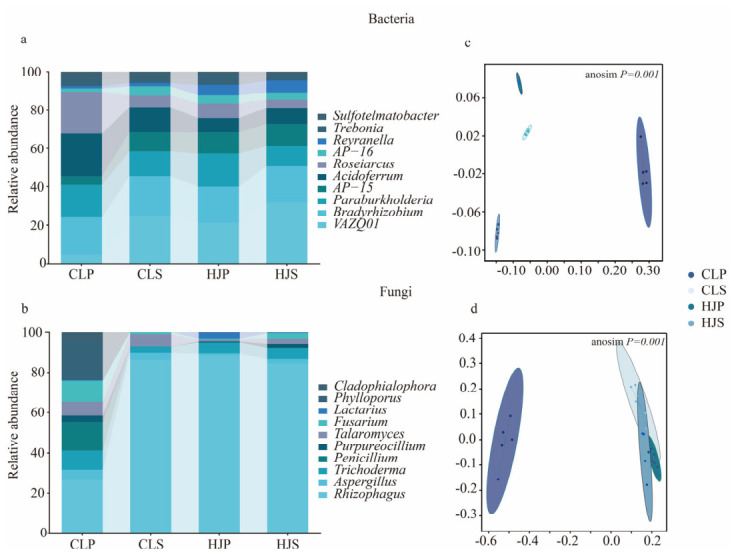
Differences in rhizosphere microbial community composition among different forest types. Note: (**a**) Bacterial community composition at genus level; (**b**) fungal community composition at genus level; (**c**) principal coordinate analysis (PCoA) of bacterial community based on Bray–Curtis distance; and (**d**) principal coordinate analysis (PCoA) of fungal community based on Bray–Curtis distance. CLP: *P. massoniana* pure forest; CLS: *S. superba* pure forest; HJP: *P. massoniana* rhizosphere in mixed forest; HJS: *S. superba* rhizosphere in mixed forest.

The taxonomic annotation results revealed that *Bradyrhizobium* (10.69–14.56%), *Paraburkholderia* (6.94–12.64%), and *AP-15* (4.96–12.81%) were the dominant bacterial taxa in the rhizosphere across all stand types, acting as the core microbes driving community structure and function (Figure 2a,b). *AP-15* and *Bradyrhizobium* exhibited the highest relative abundances in the CLP and CLS, respectively, whereas *Paraburkholderia* and *Burkholderia* were more enriched in the mixed plantations. The fungal communities were dominated by *Rhizophagus* (14.84–88.81%), *Trichoderma* (1.37–11.30%), and *Phylloporus* (11.30–16.78%). Notably, *Rhizophagus* accounted for a higher proportion in the mixed stands and CLS, whereas Trichoderma dominated in the CLP, reflecting the selective filtering of fungal communities by different stand types. The significant increase in *Paraburkholderia* in the mixed stands suggests that the introduction of *S. superba* markedly altered the rhizosphere microbiome of *P. massoniana* and enhanced the colonization of *Paraburkholderia*. A principal coordinate analysis (PCoA) demonstrated that the stand type significantly influenced both the bacterial (R^2^ = 0.935) and fungal (R^2^ = 0.8631) community structures in the rhizosphere, with a distinct separation observed between the mixed and pure plantations (Figure 2c,d).

### 2.3. Analysis of Rhizosphere Microbial Nutrient Cycling Processes and Functional Genes

#### 2.3.1. Changes in Functional Gene Abundance

**Figure 3:**
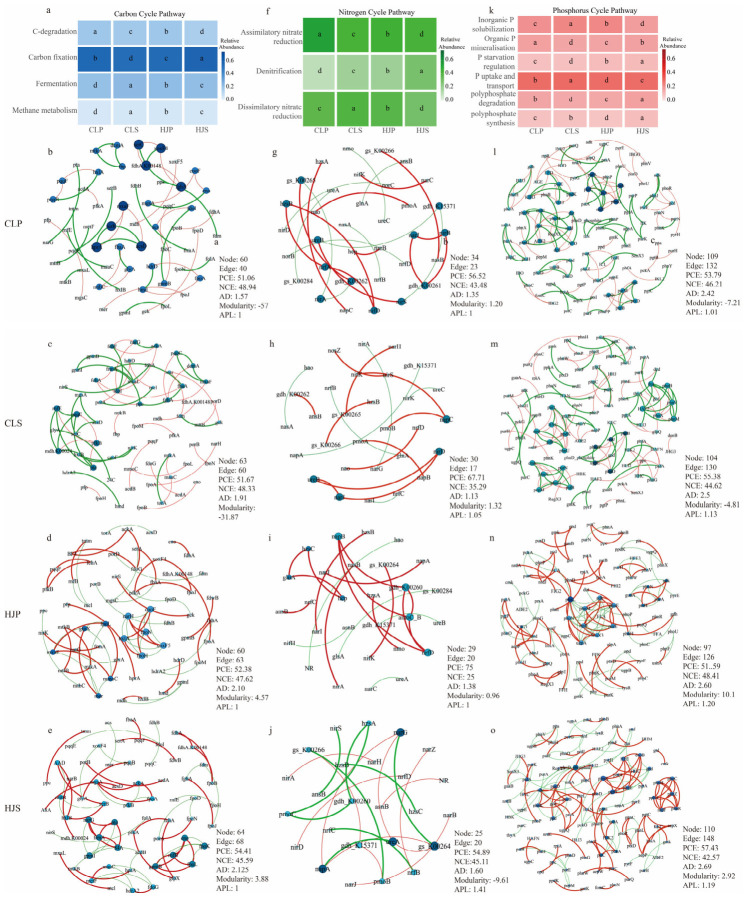
Rhizosphere microbial carbon, nitrogen, and phosphorus cycling processes and functional gene co-occurrence networks among different forest types. Note: (**a**) Carbon cycling processes; (**b**–**e**) carbon-cycling gene networks; (**f**) nitrogen cycling processes; (**g**–**j**) nitrogen-cycling gene networks; (**k**) phosphorus cycling processes; (**l**–**o**) phosphorus-cycling gene networks. PCE, positive correlation edge; NCE, negative correlation edge; AD, average degree; APL, average path length. Different letters indicate significant differences among treatments at *p* < 0.05. In graph, red lines represent positive correlations, while green lines represent negative correlations. CLP: *P. massoniana* pure forest; CLS: *S. superba* pure forest; HJP: *P. massoniana* rhizosphere in mixed forest; HJS: *S. superba* rhizosphere in mixed forest.

Mixed planting significantly altered the abundance patterns of microbial-driven carbon, nitrogen, and phosphorus cycling processes (Figure 3a,f,k). Primarily, the mixed plantations maintained higher potentials for denitrification and phosphorus starvation response regulation, with their gene abundances being significantly higher than those in the two pure stands. Specifically, denitrification and phosphorus starvation regulation in the HJP were 86.71% and 11.46% higher than in the CLP, respectively; in the HJS, denitrification, phosphorus starvation regulation, polyphosphate degradation, and polyphosphate synthesis were 59.24%, 18.84%, 16.73%, and 12.48% higher than in the CLS, respectively. Conversely, the two pure stands exhibited distinct functional differentiation: the CLS showed advantages in promoting carbon degradation, assimilatory nitrate reduction, and inorganic phosphorus solubilization, whereas the CLP demonstrated a stronger functional potential in assimilatory nitrate reduction, organic phosphorus mineralization, polyphosphate degradation, and polyphosphate synthesis.

#### 2.3.2. Changes in Network Topological Properties

The co-occurrence network analysis of functional genes related to nutrient cycling indicated that the mixed plantations exhibited significant advantages in ecological network optimization (Figure 3b–e,g–j,l–o). Mixed planting significantly increased the modularity index of carbon and phosphorus cycling networks, reshaping the homogeneously diffused structure of pure stands into a highly cohesive, modular structure. The positive correlation proportion of the nitrogen cycling network in the HJP reached as high as 75.0% (Figure 3i). Furthermore, the mixed stands displayed a higher average degree; for instance, the average degree of the phosphorus cycling network in the HJS (2.691) was significantly higher than that in the CLS (2.5), and the HJP also exhibited tighter node connectivity in both its carbon and nitrogen cycling networks.

#### 2.3.3. Ecological Interpretation of Topological Changes

The shift toward positive associations—particularly the high proportion of positive edges in the mixed stands (e.g., 75.0% in the HJP nitrogen network)—indicates a transition from resource competition (characteristic of pure stands) to synergistic metabolism (mixed stands). Such a topology, dominated by positive associations, suggests that the rhizosphere microbiome in mixed plantations shifts from a resource-competition mode to a synergistic metabolism mode, profoundly enhancing the overall efficiency of nitrogen cycling and conferring greater resilience to environmental disturbances.

### 2.4. Correlation Analysis Among Soil Properties, Enzyme Activities, Microbial Biomass, and Nutrient Cycling Processes

**Figure 4:**
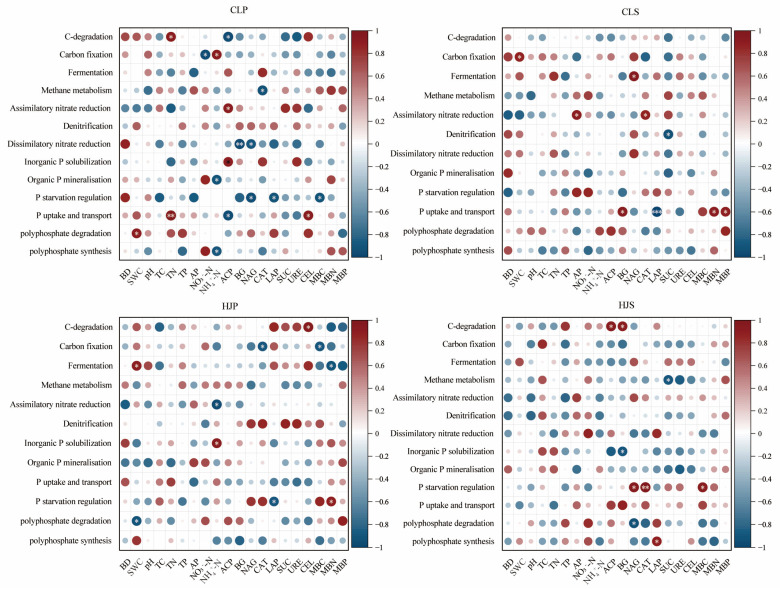
Correlation analysis between soil properties and rhizosphere microbial nutrient cycling processes. Note: BD: bulk density; SWC: soil water content; TC: total Carbon; TN: total nitrogen; TP: total phosphorus; AP: available phosphorus; NO_3_^−^-N: nitrate nitrogen; NH_4_^+^-N: ammonium nitrogen; ACP: acid phosphatase; BG: β-glucosidase; NAG: N-acetyl-β-D glucosaminidase; CAT: catalase; LAP: leucine aminopeptidase; SUC: sucrase; URE: urease; CEL: cellulase; MBC: microbial biomass carbon; MBN: microbial biomass nitrogen; MBP: microbial biomass phosphorus. *, *p* < 0.05; **, *p* < 0.01; ***, *p* < 0.001. CLP: *P. massoniana* pure forest; CLS: *S. superba* pure forest; HJP: *P. massoniana* rhizosphere in mixed forest; HJS: *S. superba* rhizosphere in mixed forest.

The correlation analysis revealed that within the carbon cycle, mixed planting significantly enhanced the positive correlations between rhizosphere microbial C cycling processes and both C cycling-related enzymes and MBC in the bulk soil (Figure 4). Within the nitrogen cycle, the mixed plantations showed strengthened negative correlations of denitrification, anammox, and nitrogen fixation with pH, TN, NO_3_^−^-N, and URE. In the phosphorus cycle, the mixed forests had significantly enhanced negative correlations among phosphorus uptake and transport, the regulation of phosphorus starvation responses, and ACP and MBP. This indicates that mixed-species models, through interspecific synergy, not only strengthen the positive coupling between the carbon cycle and the enzyme–microbial carbon system, but also significantly enhance the negative feedback regulation mechanism of the nitrogen and phosphorus cycles through multidimensional pathways involving soil physicochemical properties, enzymes, and microorganisms.

### 2.5. Redundancy Analysis (RDA) and Structural Equation Modeling (SEM)

**Figure 5:**
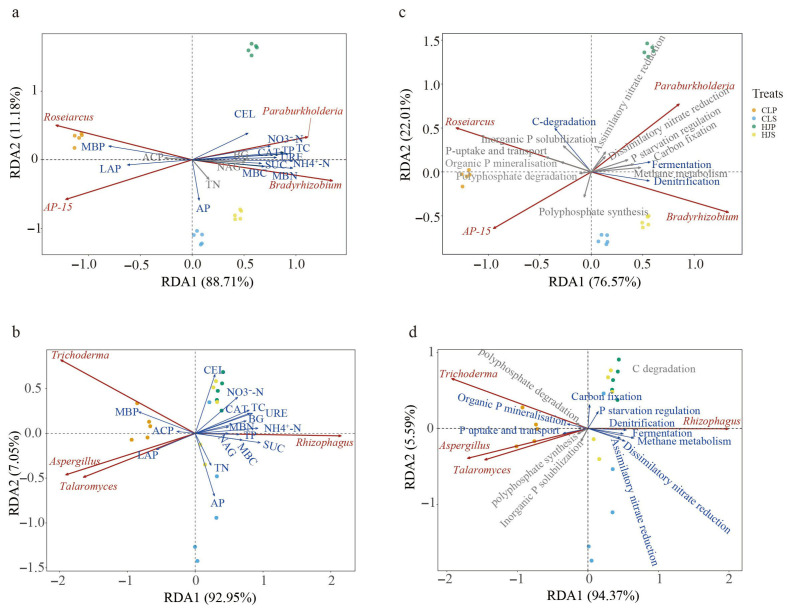
Redundancy analysis (RDA) of soil properties with microbial communities and nutrient cycling processes. Note: (**a**) RDA of soil properties and bacterial community (genera with relative abundance > 5%); (**b**) RDA of soil properties and fungal community (genera with relative abundance > 5%); (**c**) RDA of nutrient cycling processes and bacterial community; (**d**) RDA of nutrient cycling processes and fungal community. Red arrows represent significant environmental factors (*p* < 0.05). TC: total carbon; TN: total nitrogen; TP: total phosphorus; AP: available phosphorus; NO_3_^−^-N: nitrate nitrogen; NH_4_^+^-N: ammonium nitrogen; ACP: acid phosphatase; BG: β-glucosidase; NAG: N-acetyl-β-D glucosaminidase; CAT: catalase; LAP: leucine aminopeptidase; SUC: sucrase; URE: urease; CEL: cellulase; MBC: microbial biomass carbon; MBN: microbial biomass nitrogen; MBP: microbial biomass phosphorus.

The first two axes of the RDA at the genus level explained 99.89% and 99.89% of the relationships between the soil physicochemical properties, enzyme activities, microbial biomass, and the bacterial and fungal communities (relative abundance > 5%), respectively (Figure 5a,b). They also explained 98.58% and 88.71% of the variance between the soil microbial C, N, and P cycling processes and the bacterial and fungal communities, respectively (Figure 5c,d). Among the soil environmental factors, TC, AP, NO_3_^−^-N, NH_4_^+^-N, CAT, SUC, MBC, and MBN exerted significant effects on both the bacterial and fungal communities (*p* < 0.05). Additionally, the bacterial community was significantly regulated by URE, CEL, denitrification, and methane metabolism, while the fungal community was significantly influenced by TP and NAG. During nutrient cycling, carbon degradation, methane metabolism, and denitrification were the dominant factors driving the soil bacterial community, with methane metabolism and denitrification showing significant regulatory effects; meanwhile, carbon fixation, denitrification, and methane metabolism were the primary drivers for the soil fungal community.

**Figure 6:**
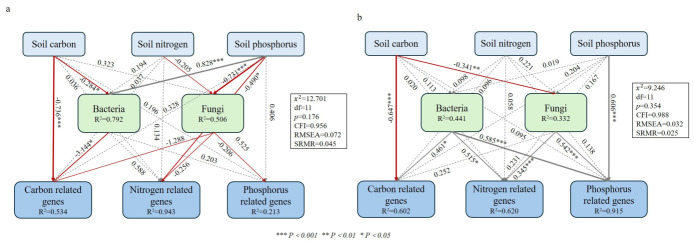
Structural equation models (SEMs) showing effects of pure and mixed forests on soil properties and functional genes. Note: (**a**) Pure forest model (integrating CLP and CLS data); (**b**) mixed forest model (integrating HJP and HJS data). Gray lines represent positive correlations, while red lines represent negative correlations. Solid lines indicate significant paths (*p* < 0.05); dashed lines indicate non-significant paths. Numbers on paths are standardized path coefficients. For model identification, all displayed paths are freely estimated in models; no paths are constrained to specific values. R^2^ values represent proportion of variance explained for endogenous variables. Model fit indices: χ^2^/df, CFI (comparative fit index), RMSEA (root mean square error of approximation).

The structural equation modeling (SEM) analysis demonstrates that the mixed plantations significantly strengthened the microbial-driven mechanisms of the soil phosphorus cycle, with the explained variance (R^2^) of phosphorus cycling genes leaping from 0.213 in the pure stands to 0.915 in the mixed stands (Figure 6). In the mixed plantations, the fungi established highly significant positive path correlations with both the nitrogen and phosphorus cycling genes (r = 0.345, r = 0.542, *p* < 0.001), paths that were entirely non-significant in the pure stands, confirming that fungi are the core force driving phosphorus turnover in mixed systems. The soil phosphorus exerted a highly significant direct positive effect on the phosphorus cycling genes (path coefficient = 0.606, *p* < 0.001), and the fungal community also had a highly significant direct positive effect on these genes (path coefficient = 0.542, *p* < 0.001). This indicates that fungi (primarily the arbuscular mycorrhizal fungus *Rhizophagus*) act as a core biological group independently driving the expression of phosphorus cycling genes in mixed plantations. Through an indirect pathway where soil phosphorus promotes fungal enrichment and fungi subsequently directly regulate phosphorus cycling genes, mixed planting shifts the driving force of the phosphorus cycle from direct environmental stress (in pure stands) to synergistic biological regulation, while fungi simultaneously play a critical role in the nitrogen cycle.

## 3. Discussion

### 3.1. Mechanisms of Soil Property Improvement by Mixed Planting

In this study, the mixed planting of *P. massoniana* and *S. superba* significantly increased the contents of soil organic carbon, NO_3_^−^-N, and NH_4_^+^-N, as well as the activities of key hydrolytic enzymes, such as SUC, CEL, and URE. These improvements can largely be attributed to interspecific niche complementarity and synergistic effects, as reported in previous studies [20,21]. As a broadleaved species, *S. superba* produces litter with a lower C/N ratio; along with its diverse root exudates, it provides higher-quality carbon and energy sources for soil microorganisms. This, in turn, markedly stimulates microbial activity and accelerates organic matter decomposition and nitrogen mineralization [22,23,24]. Compared with the relatively homogeneous litter input in pure stands, mixed plantations introduce greater substrate heterogeneity through diversified inputs, thereby establishing a richer resource foundation for functionally diverse microbial communities [1] and enhancing the utilization efficiency of organic carbon, nitrogen, and phosphorus. Moreover, the widespread increase in enzyme activities indicates more active microbial metabolic processes, supporting the notion that mixed plantations can accelerate organic matter decomposition [25,26]. This enhancement in enzyme activity further promotes soil nutrient turnover, especially in carbon and nitrogen cycles, ultimately improving soil fertility.

Additionally, mixed planting resulted in a significant increase in the microbial biomass carbon and nitrogen, as illustrated in Figure 2. This directly reflects the enhanced heterogeneity of rhizosphere substrates, which provides more abundant energy and nutrients to support microbial growth [24,27]. The introduction of *S. superba* alleviated the long-standing carbon and nitrogen limitations typical of pure *P. massoniana* plantations by contributing litter with a low C/N ratio, thereby facilitating microbial biomass accumulation. In contrast, the microbial biomass phosphorus in the mixed plantations was significantly lower than that in the pure pine stands. This contrast indicates a fundamental shift in the microbial phosphorus utilization strategy: in pure pine stands, severe phosphorus deficiency and low availability drive microorganisms to immobilize the limited phosphorus within their own biomass [28,29]. However, in mixed planting, the increased availability of phosphorus, along with its efficient mobilization by specific functional taxa, such as *Paraburkholderia* and mycorrhizal fungi like *Rhizophagus*, prompt microorganisms to shift away from biological immobilization and to accelerate phosphorus turnover within the soil microbe plant system. This transition from immobilization to efficient turnover serves as a key biological mechanism that enables mixed plantations to overcome phosphorus limitation in the red soils of southern China [30,31].

### 3.2. Effects of Mixed Planting on Rhizosphere Microbial Community Structure

Mixed planting induced a profound taxonomic reshaping of the rhizosphere microbiome, driven by interspecific interactions and altered soil microenvironments [31,32]. Within the bacterial community, the introduction of *S. superba* favored the significant enrichment of specific genera, most notably Paraburkholderia and Burkholderia, which emerged as core members of the mixed plantation rhizosphere. As the dominant broadleaved tree species, *S. superba* likely exerted a strong selective pressure through its diverse root exudates, selectively filtering and recruiting these distinct bacterial taxa [33,34].

Concurrently, the fungal community experienced a dramatic shift in dominance. The arbuscular mycorrhizal fungus Rhizophagus became overwhelmingly dominant in the mixed plantation rhizosphere, with its relative abundance surging to range between 14.84% and 88.81%. In contrast, Trichoderma dominated in the pure pine stands, reflecting a clear selective filtering of fungal assemblages by stand type [19,35]. The redundancy analysis (RDA) confirms that environmental factors are the core drivers shaping these compositional shifts. By significantly raising the levels of total carbon, NO_3_^−^-N, and available phosphorus, mixed plantations alleviate native resource constraints and steer the community structure toward distinct assembly patterns [36,37]. Notably, the fungi and bacteria exhibited a clear niche differentiation in their responses to soil phosphorus: the fungal community structure was primarily shaped by the total phosphorus, whereas the bacterial community responded more directly to the available phosphorus. This taxonomic differentiation laid the necessary structural foundation for the functional complementarity observed in the mixed ecosystem [38,39].

### 3.3. Effects of Mixed Planting on C-N-P Nutrient Cycling Functions and Ecological Coupling Mechanisms

Building upon this taxonomically reshaped microbiome, mixed planting profoundly alters the functional potentials and network interactions governing soil C-N-P cycling. The co-occurrence network analysis of functional genes reveals a fundamental shift in microbial metabolism: from a resource-competitive mode characteristic of pure stands toward a highly synergistic mode in mixed stands. The average degree, modularity index, and the proportion of positive correlations are all significantly higher in the mixed plantations. Notably, the proportion of positive correlations in the nitrogen cycling network reaches 75.0%, indicating the formation of a synergy-dominated ecological network [40,41,42].

This observed structural shift from a competitive to a synergistic functional network is likely driven by several interacting mechanisms. First, the chemically diverse root exudates from mixed-species stands provide a highly heterogeneous carbon pool, which promotes metabolic specialization (reflected in the higher modularity) and microbial cross-feeding (driving the increase in positive correlations). Second, the alleviated nutrient limitation—such as the reduced phosphorus immobilization observed in mixed stands—likely lowers the interspecific competitive pressure, allowing for cooperative traits to emerge and stabilize within a community. Finally, the keystone mutualistic taxa, such as arbuscular mycorrhizal fungi, may physically and functionally interconnect spatially separated nutrient patches via their extensive hyphal networks, fundamentally fostering overall network cohesion and inter-microbial synergy [43,44,45].

The structural equation modeling (SEM) provides direct causal evidence for this biological synergy. The model reveals that mixed planting significantly strengthens the microbially driven mechanisms of the soil phosphorus cycle, with the explained variance (R^2^) of P cycling genes leaping from 0.213 in the pure stands to 0.915 in the mixed stands. In this synergistic framework, fungi—specifically keystone taxa like *Rhizophagus*—transition into the core biological drivers that independently upregulate the expression of phosphorus cycling genes (path coefficient = 0.542, *p* < 0.001). Rather than merely persisting as abundant taxa, these fungi act as central functional hubs that actively reshape biogeochemical pathways. Through this indirect pathway where soil phosphorus availability promotes fungal enrichment, and fungi subsequently drive functional gene expression, mixed planting transforms the soil phosphorus pool from a competitive sink into a dynamic, efficiently cycling source, effectively breaking the nutrient limitation bottleneck in subtropical red soils.

## 4. Materials and Methods

### 4.1. Site Description

**Figure 7:**
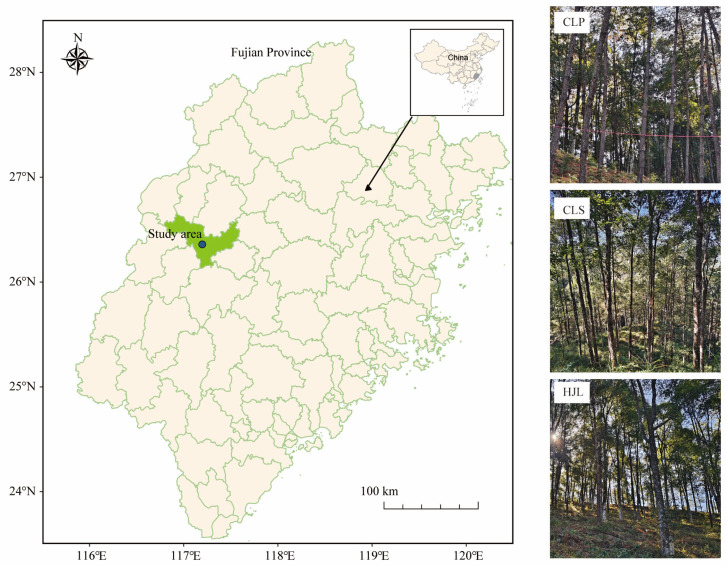
Experimental site locations and study area overview. Note: CLP: *Pinus massoniana* pure forest; CLS: *Schima superba* pure forest; HJL: mixed forest.

The study area is located in the Mingxi State-owned Forestry Farm, Sanming City, Fujian Province, China (26°21′34″ N, 117°11′49″ E) (Figure 7). This region exhibits a subtropical monsoon climate, characterized by a mean annual temperature of 18.1 °C and an average annual relative humidity of 81%. The frost-free period lasts for 283 days. The mean annual precipitation measures 1786 mm, and it receives approximately 1750.7 h of annual sunshine. The topography is predominantly mountains, with an average elevation of 517 m, and the primary soil types are red and yellow soils. The understory vegetation is primarily composed of species such as *Mallotus apelta*, *Ilex pubescens*, and *Dicranopteris dichotoma*.

### 4.2. Experimental Design and Stand Inventory

In November 2024, pure *P. massoniana* plantations (CLPs), pure *S. superba* plantations (CLSs), and mixed plantations (HJLs) were selected as the subjects of this research. Within the mixed stands, individual *P. massoniana* (HJP) and *S. superba* (HJS) trees were specifically targeted for sampling. All the stands were established in 1990 at the Mingxi State-owned Forestry Farm in Fujian Province. Five replicate plots, each measuring 20 m × 20 m, were established for each of the three stand types, totaling 15 plots. A complete tree tally (including tree height and diameter at breast height, DBH) was conducted within each plot. The tree growth characteristics for each stand type are presented in Table 1.

### 4.3. Soil Sampling

Bulk soil sampling: Within each plot, an “S-shaped” five-point sampling method was employed to collect soil from the 0 to 20 cm layer after the removal of surface litter. The collected soil samples were placed in ice boxes and transported to the laboratory. After carefully removing visible roots, stones, and other debris, the soil was passed through a 2 mm sieve [46]. The sieved soil was then divided into two subsamples: one was air-dried naturally for the determination of pH, total carbon (TC), total nitrogen (TN), and total phosphorus (TP), while the other was stored at 4 °C for the analysis of ammonium nitrogen (NH_4_^+^-N), nitrate nitrogen (NO_3_^−^-N), available phosphorus (AP), enzyme activities, and microbial biomass.

Rhizosphere soil sampling: In each plot, 8–10 target trees with similar growth conditions and diameter at breast height (DBH) were randomly selected. Surface vegetation and impurities were removed using a spade, and the soil was carefully excavated to locate the fine roots (diameter ≤ 2 mm) of the trees. After manually shaking off the loosely adhering bulk soil, the fine roots with the remaining attached soil were placed into sterile centrifuge tubes containing 10 mL of sterile phosphate-buffered saline (PBS). The tubes were vortexed at 2500 rpm for 5 min to detach the tightly adhering soil particles. The roots were then removed using sterile forceps, and the remaining suspension was centrifuged at 4000× *g* for 10 min. The supernatant was discarded, and the pellet was collected as the rhizosphere soil sample. The rhizosphere soil from the same tree species within a plot was pooled into a single composite sample. These composite samples were sealed in sterile plastic bags, immediately placed in an insulated cooler (below 0 °C) for transport, and subsequently stored at −80 °C prior to metagenomic sequencing.

### 4.4. Soil Physicochemical and Biological Analyses

Soil physicochemical properties were determined using standard analytical methods as described by [47,48,49]. Briefly, soil pH was measured using glass electrode, while total carbon (TC), total nitrogen (TN), and total phosphorus (TP) were quantified using dichromate oxidation, Kjeldahl digestion, and molybdenum–antimony anti-spectrophotometry, respectively. Available nutrients (AP, NH_4_^+^-N, and NO_3_^−^-N) were extracted and analyzed using continuous flow analysis. Soil enzyme activities (BG, NAG, LAP, ACP, SUC, CEL, URE, and CAT) were determined via fluorometric microplate assays and conventional colorimetry. Soil microbial biomass carbon, nitrogen, and phosphorus (MBC, MBN, and MBP) were quantified using chloroform fumigation–extraction method.

The soil metagenomic sequencing and data preprocessing genomic DNA were extracted from the collected rhizosphere soil samples using a FastDNA™ SPIN Kit for Soil (MP Biomedicals, Solon, OH, USA). The purity and integrity of the extracted DNA were evaluated via agarose gel electrophoresis (AGE), and the DNA concentration was accurately quantified using a Qubit fluorometer (Thermo Fisher Scientific, Waltham, MA, USA). The qualified DNA samples were fragmented to approximately 350 bp using a Covaris ultrasonic disruptor (Covaris, Woburn, MA, USA). Metagenomic sequencing libraries were subsequently constructed through a standard workflow, including end repair, A-tailing, sequencing adapter ligation, purification, and PCR amplification. Shotgun metagenomic sequencing was performed on an Illumina high-throughput sequencing platform with a paired-end (PE150) strategy. The raw sequencing data were subjected to strict quality control to remove low-quality reads and adapter sequences, yielding high-quality clean data. Subsequently, the clean reads were de novo assembled, followed by gene prediction and dereplication to construct a non-redundant gene catalog. Finally, the high-quality reads from each sample were mapped back to the non-redundant gene catalog to calculate the abundance of each gene [50,51,52].

Taxonomic profiling and functional gene annotation: The amino acid sequences from the non-redundant gene catalog were aligned against the NCBI non-redundant protein (NR) database and the KEGG database (version 2021) using DIAMOND software ((version 2.0.15. BLASTP, with an expected value threshold set to 1 × 10^−5^). Based on the alignment results, the relative abundances of microbial taxonomic composition and functional profiles at various levels were determined. Concurrently, the target functional genes associated with the major carbon (C), nitrogen (N), and phosphorus (P) cycling pathways identified in the metagenomes were filtered. The functional genes involved in C cycling were categorized into four groups: carbon degradation, carbon fixation, fermentation, and methane metabolism. The functional genes involved in N cycling were categorized into four groups: anammox, assimilatory nitrate reduction, denitrification, and nitrogen fixation. Furthermore, the functional genes involved in P cycling were categorized into six groups: inorganic phosphorus solubilization, organic phosphorus mineralization, phosphorus starvation response regulation, phosphorus uptake and transport, polyphosphate degradation, and polyphosphate synthesis.

### 4.5. Statistical Analysis

One-way analysis of variance (ANOVA) was used to compare soil physicochemical properties, enzyme activities, microbial biomass, and functional gene abundances across stand types (CLP, CLS, and HJL). Prior to ANOVA, normality was assessed using Shapiro–Wilk test, and homogeneity of variances was verified using Levene’s test. When assumptions were violated, data were log-transformed; if normality or homoscedasticity could not be achieved after transformation, non-parametric Kruskal–Wallis test was applied. Following significant ANOVA (or Kruskal–Wallis), post hoc comparisons were conducted using Tukey’s honestly significant difference (HSD) test (α = 0.05). Principal coordinate analysis (PCoA) based on Bray–Curtis distance matrices was performed to visualize differences in bacterial and fungal community composition among treatments. Permutational multivariate analysis of variance (PERMANOVA) with 9999 permutations was used to test significance of stand-type effects on community structure. Redundancy analysis (RDA) was conducted to identify key environmental factors (soil properties, enzyme activities, and microbial biomass) driving variations in microbial community composition and functional gene profiles. Significance of explanatory variables was tested using 999 permutations. Co-occurrence networks of carbon, nitrogen, and phosphorus cycling genes were constructed based on Spearman’s rank correlation coefficients. Only robust and statistically significant correlations (|r| > 0.6, *p* < 0.01) were retained. Network topological parameters—including average degree (AD), average path length (APL), modularity, and proportion of positive vs. negative edges—were calculated using igraph package. Networks were visualized with Gephi (version 0.9.2). SEM was performed using lavaan package (version 0.6-12) in R. Models were constructed a priori based on established ecological theories: soil properties (TC, TN, TP, AP, NO_3_^−^-N, and NH_4_^+^-N) as exogenous variables, microbial community composition (relative abundances of dominant bacterial and fungal genera) as mediating variables, and functional gene abundances (C-cycling, N-cycling, and P-cycling gene groups) as endogenous variables. Separate models were fitted for pure forest data (CLP + CLS) and mixed forest data (HJP + HJS). Model fit was assessed using following criteria: χ^2^/df < 3.0, comparative fit index (CFI) ≥ 0.90, and root mean square error of approximation (RMSEA) ≤ 0.08. All paths were freely estimated; no post hoc modifications were made to initial models. Path coefficients are reported as standardized values.

## 5. Conclusions

Our study demonstrates that mixing *P. massoniana* with *S. superba* fundamentally transforms soil nutrient cycling from a resource-competitive mode—characteristic of pure stands—to a microbially synergized mode. In pure stands, nutrient cycling is constrained by microbial competition for limited resources, resulting in functional gene networks dominated by negative correlations and inefficient phosphorus (P) cycling (R^2^ = 0.213). Conversely, mixed planting establishes a synergistic network characterized by high modularity, an overwhelming proportion of positive interactions (e.g., 75.0% in the nitrogen cycling network), and fungal-driven P mobilization (path coefficient = 0.542, R^2^ = 0.915). This rhizosphere microbial effect—defined here as the structural shift from competitive to synergistic functional gene interactions—is the critical internal mechanism driving soil quality improvement. We propose that this rhizosphere microbial effect serves as a powerful diagnostic indicator of the recovery of degraded pine forests. Specifically, the network topological parameters—such as an average degree ≥ 2.5, a positive correlation ratio ≥ 70%, and significantly elevated modularity indices relative to pure stands—can function as early warning signals of restoration success. By capturing the emergent properties of the microbial community, these metrics offer substantially more information than conventional single-parameter indicators, such as isolated enzyme activities or gene abundances. Consequently, the rhizosphere microbial effect identified here, evidenced by robust network connectivity and functional gene synergy, serves as more than just a mechanism for soil health improvement. We propose that these specific microbial network metrics (e.g., modularity, positive interaction ratios) and synergy signatures can be utilized as practical, early diagnostic indicators for evaluating soil recovery trajectories. Applying these ecological network indicators will provide targeted, quantifiable benchmarks for assessing the success of ecological restoration and close-to-nature management of degraded pine forest systems.

## Figures and Tables

**Table 1:** Tree growth characteristics of different forest types (mean ± standard deviation).

Forest Type	Species	Tree Height/m	Diameter at Breast Height/cm
CLP	*Pinus massoniana*	17.88 ± 1.22 b	18.47 ± 1.03 b
CLS	*Schima superba*	13.44 ± 1.18 a	13.44 ± 1.10 a
HJL	*Pinus massoniana* (HJP)	19.46 ± 0.69 a	20.47 ± 2.31 a
	*Schima superba* (HJS)	14.92 ± 1.12 a	13.94 ± 0.98 a

Note: CLP: *Pinus massoniana* pure forest; CLS: *Schima superba* pure forest; HJL: mixed forest; HJP: *P. massoniana* in mixed forest; HJS: *S. superba* in mixed forest. Values are means ± standard deviation. Different lowercase letters within the same species indicate significant differences between pure and mixed forests at *p* < 0.05; data are presented as means ± standard deviation.

## Data Availability

The data presented in this study are available on request from the corresponding author.
